# The Book World of Medicine and Science

**Published:** 1898-10-15

**Authors:** 


### The Book World of Medicine and Science.

The Determination of Sex. By Dr. Leopold Schenck, Professor and Director of the Embryological Institute, at the University of Vienna. (London: 1898. The Werner Company. Pp. 173, 8vo.)

Now that the wonder-seeking daily press has done with this subject to feast its scientific appetite on tales "of anthropophagi and men whose heads do grow beneath their shoulders," a time has come when Professor Schenck's remarkable theory may well be submitted to more sober criticism. It must be remembered in the first place that Professor Schenck is a man of great distinction and position in science, an acknowledged master in his own branch. His deliberately expressed opinion cannot, therefore, be pooh-poohed or dismissed summarily without investigation. It behoves us rather to carefully examine his arguments and the practical conclusions to which they lead.

The book opens with a summary of the various theories which have been put forward as to the predetermination of sex. Many of these are fanciful, some absurd, but a fair amount of evidence is adduced in favour of the view that the worse-fed parent tends to perpetuate itself. On this is based the theory of sexual cross-heredity of which Professor Schenck afterwards makes use. This theory was further elaborated by Thury, who came to the conclusion that the sole cause of sex lies in the ovum developing itself in the ovary, the degree of its ripeness being the only factor in the development of one sex or the other. Professor Schenck epitomises the researches of Thury and his successors, but we can hardly regard this part of the book as satisfactory, the various abstracts being given without any systematic arrangement, and with, practically speaking, no attempt at criticism. One feels also that exact bibliographical references would be preferable to the mere mention of the names of authors.

The second section opens with the statement that Professor Schenck, having considered the various views enumerated, finds himself compelled to fall back upon the theory of the cross-heredity of sex and to endeavour to establish it by experiments of his own. To this end he considers the examination of the excreta of supreme importance; his work has to do in particular with the sugar found in normal urine. Using the phenyl-hydrazine test he assumes that the absence of a reaction indicates perfect metabolism, all the carbohydrates being completely burnt up, while its presence means a slight metabolic imperfection which, although insufficient to cause ill-health, is enough to render a woman liable to produce a girl, whereas the perfect metabolic balance would by the law of crossed heredity ensure the birth of a boy. His practical conclusion is that if one wishes to determine male offspring the mother must be dieted so as to cause the disappearance of this normal urine-sugar, as he calls it. He quotes a few striking cases in which he claims to have done this with the desired effect, and concludes that by his system of dieting a woman can become sexually superior to a man, and her offspring will accordingly be male.

Such is the theory which has caused so much general interest, and aroused such a howl of execration among Professor Schenck's colleagues at Vienna. We cannot here discuss it in all its details, but we feel compelled to suggest that an inherent weakness seems to be the assumption that one parent alone is concerned in determining the sex of conjoint offspring. Should this contention not be upheld, it is obvious that the male factor will also have to be reckoned with and treated, a point which the author does not consider at all. Furthermore, we should like to know the logical process by which Professor Schenck came to the conclusion that the carbohydrates of the urine are to be regarded as the sole guide to the physical condition of an apparently normal individual. These objections may not be insuperable, but they certainly demand consideration, and though we cannot join in the chorus of critics who wish to brand the author as a fool or an impostor, we feel that, without further substantiation, we are bound to view his theory with grave scepticism.

First Aid to the Injured. By E. T. Lawless, M.D., Surgeon-Major 4th V.B. East Surrey Regiment. Pp. 201. Illustrated. Price 3s. 6d. (London: The Scientific Press, Ltd. 1898.)

This book is, as the publishers' note states, a reprint without alteration of Dr. Lawless' well-known work. There is little need, therefore, to indicate its scope and general plan, as these must be tolerably familiar. Suffice it to say, then, that all the information considered necessary for bearers in the field, from elementary anatomy to stretcher drill and the making of field kitchens, is here supplied. While therefore an excellent basis is given for popular lectures on first aid, there is much additional matter that Volunteer surgeons will find it necessary to be acquainted with. The book, in fact, follows the lines of the official manual for the Medical Staff Corps; and Dr. Lawless has succeeded in clothing the very dry bones of that excellent though arid work with living flesh. The chapter on "Health, and its Preservation in Camp," is, for example, an excellent instance of the advantages of "unofficial" treatment of such a subject. But we cannot forbear calling attention to the omission of any attempt to explain the phenomena of wound infection, and the paramount necessity for aseptic or antiseptic treatment of wounds. It is true that in one place the use of "antiseptic water" is advised. But little more is said other than that "Carbolic water (1 in 40 or 100) is the most common antiseptic water." If it were not for this we should have nothing but praise for Surgeon-Major Lawless' manual.

Von Gudthausen's Sprachführer fur die Ärzsliche und Pharmaceutische Praxis. (German-French. Arthur Georgi, Leipzig.)

This is a handy little dictionary of medical terms and expressions for those engaged in medical and pharmaceutical practice who are unfamiliar with the French equivalents. The author has followed the same lines as those employed in the German-English portion, that is to say it is divided into seventeen sections—the human body; diseases, sickness, and wounds; dressings and ambulances, &c. As a book of reference it is possible that information would have been more easily acquired if the sections had been omitted and a simple alphabetical order adhered to. For students anxious to learn the equivalents the plan adopted is the best. As a precise and scientific work we should have preferred to have seen the Latin type employed: with the death of Bismarck the German is almost an anachronism. In other respects the dictionary is deserving of all praise—accurate, precise, and practical.

